# Expression and Significance of Matrix Metalloproteinase-2 and Matrix Metalloproteinas-9 in Endometriosis

**DOI:** 10.25122/jml-2020-0117

**Published:** 2020

**Authors:** Adrian Mykhailovych Barbe, Andrii Mykolaiovych Berbets, Igor Sviatoslavovych Davydenko, Halyna Danylivna Koval, Viktoriia Oleksandrivna Yuzko, Oleksandr Mykhailovych Yuzko

**Affiliations:** 1. Department of Obstetrics and Gynecology, Bukovinian State Medical University, Chernivtsi, Ukraine; 2. Department of Pathologic Anatomy, Bukovinian State Medical University, Chernivtsi, Ukraine; 3. Department of Clinical Immunology, Allergology and Endocrinology, Bukovinian State Medical University, Chernivtsi, Ukraine

**Keywords:** Endometriosis, MMP-2, MMP-9, metalloproteinase, neo-angiogenesis, cellular matrix, CA-125 – carbohydrate antigen-125, DAB – Diaminobenzidine, HE4 – human epididymis protein 4, HRP – horseradish peroxidase, IHC – immunohistochemistry, MMP – matrix metalloproteinase, TIMP – tissue inhibitors of metalloproteinase, VEGFR – vascular endothelial growth factor receptor

## Abstract

Endometriosis is a chronic benign hormone-dependent condition when the endometrial tissue, identical with the endometrium by its morphological and functional properties, grows outside the borders of the uterine mucous membrane. Recent studies have pointed to the possible role of matrix metalloproteinases (MMPs) in the pathogenesis of endometriosis. We suggested a hypothesis that increased expression of MMPs activity in eutopic and ectopic endometrium of patients with endometriosis might correlate with the presence of endometriotic lesions.

The aim of the study was to evaluate the level of MMP-2 and MMP-9 expression in the ectopic endometrium of women with visible endometriotic lesions and eutopic endometrium in patients with no signs of endometriosis.

The study was conducted on 43 patients. They were divided into two groups. Group 1 included 31 patients with peritoneal/ovarian endometriosis who had undergone laparoscopy and hysteroscopy. Group 2 consisted of 12 patients with leiomyoma, endometrial polyps or relatively healthy patients who had undergone hysterectomy or polypectomy and endometrial curettage.

This study showed statistically higher expression of MMP-2 (1.7783 ± 0.22 immunohistochemistry (IHC) optical density score compared to the control group – 1.41± 0.34, p = 0.0017) and MMP-9 (1.352 ± 0.067 versus 1.85 ± 0.26 in the control group, p = 0.001) in ectopic and eutopic endometrium samples from patients with endometriosis compared to samples taken from patients without endometriosis. A strong correlation between expression of the above-mentioned MMPs (r=0.74 for MMP-2 and r=0.88 for MMP-9) in ectopic and eutopic endometrium might be of promising diagnostic value.

## Introduction

Endometriosis is a chronic benign hormone-dependent condition when the endometrial tissue, identical with the endometrium by its morphological and functional properties, grows outside the borders of the uterine mucous membrane. It leads to clinical symptoms able to affect the physical condition, psychological status, and social status of the patient [1-4]. According to research data, endometriosis is diagnosed in 5-10% of the female population. There are approximately 176 million women with endometriosis in the world, mainly of reproductive age.

Nowadays, the pathogenetic theory of retrograde outflow of endometrial cells into the peritoneal cavity is gaining increasing support [5, 6]. Since retrograde menstruation occurs in many women, it is postulated that endometriosis develops as a consequence of disturbances in the balance between the amount of menstrual blood and capacity of the “clearance” system in the peritoneal milieu. Recent studies have pointed to the possible role of matrix metalloproteinases (MMPs) in the pathogenesis of endometriosis. In particular, Wenzl and Heinzl [7, 8] suggested that ectopic endometrium maintains its protease secretory capacity, which, in turn, allows the invasion of surrounding tissues and the subsequent formation of endometrial foci [8].

Matrix metalloproteinases (MMPs) are a family of endopeptidases playing a specific role in the degradation and turnover of the extracellular matrix (ECM). These zinc-dependent enzymes, including collagenases, gelatinases, and stromelysins, are capable of degrading all the ECM components. Tissue inhibitors of metalloproteinases (TIMPs), which affect normal and pathologic matrix remodeling, regulate the activity of MMPs [9, 10]. 

The implantation of endometrium cells results from imbalance and excretion of growth factors. Adequate blood supply and neo-angiogenesis play an essential role in the successful implantation and occurrence of ectopic foci [11-14]. However, for triggering this process, primarily, there is a need for endometrium cell adhesion, proteolytic activity, and extracellular matrix alteration. MMPs and their natural inhibitors – tissue inhibitors of metalloproteinases (TIMPs) play an important role in the remodeling of the endometrial tissue during the normal menstrual cycle [15, 16], as well as in endometriosis [1, 17, 18]. MMPs and TIMPs play a specific role in testicular development and maturation [19] and in the ovary during ovulation [20].

The peritoneal fluid of women with endometriosis is a specific microenvironment containing numerous substances that can act on the endometriotic tissue [21]. In particular, it contains an increased number of macrophages and their secretory products, such as growth factors, cytokines, and angiogenic factors [22]. On the other hand, increased metalloproteinase activity in the endometrium, in its turn, might facilitate the breakdown of the peritoneal extracellular matrix and establishment of endometriotic foci in the peritoneal cavity [23]. 

These observations have prompted us to suggest a hypothesis that increased expression of MMPs activity in eutopic endometrium combined with increased expression of vascular endothelial growth factor receptor 2 (VEGFR-2) in the peritoneum, eutopic and ectopic endometrium of patients with endometriosis might correlate with the presence of endometriotic lesions [24]. If this is the case, a simple endometrium biopsy might prove useful in assessing the early stages of endometriosis. 

The aim of the study was to evaluate the level of MMP-2 and MMP-9 expression in ectopic and eutopic endometrium of women with visible endometriotic lesions and eutopic endometrium in patients with no signs of endometriosis. We also examined the possible correlation between MMP-2 and MMP-9 levels in eutopic and ectopic endometrium in patients with endometriosis. As expected, our findings will provide a theoretical basis for a better understanding of the role of matrix metalloproteinases as possible early diagnostic markers of endometriosis.

## Material and Methods

### Subjects

The study was conducted on 43 patients who were treated at the Gynecological Department of Chernivtsi Municipal Clinical Maternity Hospital No. 1 and the Medical Center of Infertility Treatment (Chernivtsi, Ukraine). All the patients were premenopausal. 

They were divided into two groups. Group 1 included 31 patients with peritoneal/ovarian endometriosis who had undergone laparoscopy and hysteroscopy. Patients were selected by the existence of visible peritoneal endometriotic lesions (stage II, III) (ASRM, 1997), characterized as red- or gland-like lesions as well as red vesicles. 

The age of the patients ranged from 22 to 48 years, with an average of 33.8 years. The diagnosis of endometriosis was confirmed histologically by detecting endometrial-like glands in the heterotopic locations (peritoneum or ovarium). The patients with comorbid gynecologic diseases, including leiomyoma and endometrial polyps, were excluded.

Group 2 consisted of 12 patients with leiomyoma, endometrial polyps, or relatively healthy patients who had undergone hysterectomy or polypectomy and endometrial curettage. None of the patients had identifiable adenomyosis by ultrasonography imaging. 

All the endometrial samples were collected in proliferative menstrual cycle phases. The day of the menstrual cycle was established from the women’s menstrual history and was confirmed by endometrial dating using the criteria of Noyes et al. For each case of endometriosis, representative slides of eutopic and ectopic endometrium were selected. For cases of the control group, representative slides of eutopic endometrium in the proliferative phase were selected.

### Immunohistochemistry

MMP-2 and MMP-9 in the eutopic and ectopic endometrial tissue were identified by means of immunoassay diagnostic kits based on the antibodies specific for the mentioned antigens and receptors (manufactured by Abcam, Cambridge, United Kingdom).

Visualization of antibodies was performed by mouse-specific HRP/DAB Detection IHC Kit with diaminobenzene ink (manufactured by Abcam, Cambridge, United Kingdom). Cell nuclei were additionally stained with Mayer’s hematoxylin solution. The immunohistochemistry (IHC) optical density score assessment of immunohistochemical staining of MMP-2 and MMP-9 was performed (see below).

### Assessment of MMP-2, MMP-9 Staining

Cytoplasmic staining was defined as positive. Semiquantitative and quantitative analysis of the immunostainings for MMP-2 and MMP-9 was performed for each case. MMP-2 and MMP-9 expression was evaluated in the glandular and stromal cells. The staining of the superficial epithelium and stroma was scored according to the intensity for both MMP-2 and MMP-9. The results of staining were assessed by one highly qualified pathologist as an observer in a blind fashion.

### Image acquisition

Images were captured using the bright field technique with a binocular microscope (Olympus СХ-21).

Images were captured at ×10 magnification using color photo camera Olympus С450 attached to a computer system. The field was selected with good contrast between the DAB chromogen and hematoxylin, which is considered the region of interest. After capturing the images, the color density and white balance were standardized for all the images. All the acquired images were saved as a JPEG format with minimal compression.

### ImageJ analysis

For IHC image analysis, the 1.48 version of ImageJ (free software, NIH, Bethesda, Maryland) (Java 1.8.9) was applied. IHC profiler plugin with cytoplasmic-stained mode was used for qualifying images. Images were qualified as high positive, positive, low positive, and negative with the corresponding percentage of cells. As a result, the IHC Optical Density Score was calculated by using the following formula [25]:

IHC optical density score = (percentage contribution of high positive × 4 + percentage contribution of positive × 3 + percentage contribution of low positive × 2 + percentage contribution of negative × 1) / 100.

### Statistical Analysis

Statistical data were calculated and compared using the MedCalc software, developed by “MedCalc Software” (Ostend, Belgium).

The immunohistochemical data are reported as the mean ± SEM. The data obtained were statistically processed using the Mann-Whitney U test. Bivariate correlation between variables was determined by Pearson’s correlation coefficients. A P-value < 0.05 was considered significant. Results are represented as mean ± SD.

### Ethical approval

The study was approved by the Biological and Medical Ethics Committee of the Higher State Educational Establishment of Ukraine “Bukovinian State Medical University” (the minutes No. 6 dated September 15th, 2016). It was carried out strictly following The Code of Ethics of the World Medical Association (Declaration of Helsinki, 1983) for medical research involving human subjects, including research on identifiable human material and data.

## Results

### Patients’ characteristics

Patients’ characteristics of each of the groups studied are presented in Table 1. Our study has shown that the number of pregnancies significantly differed between the groups (0.87 in the study group vs. 1.66 in the control group), as many studies have shown before [26-28]. However, no significant differences between the studied groups were found concerning age, number of childbirths, number of abortions and number of miscarriages.

**Table 1:** Patients’ characteristics of the studied groups.

	Group 1, n=31	Control group, n=12	p
**Age, years**	32.61	37.00	0.07
**Number of pregnancies**	0.87	1.66	0.022
**Number of childbirths**	0.77	1.25	0.139
**Number of abortions**	0	0	-
**Number of miscarriages**	0.06	0.16	0.35

Note: The values are expressed as mean (SD).

### MMP-2 expression

**Figure 1:**
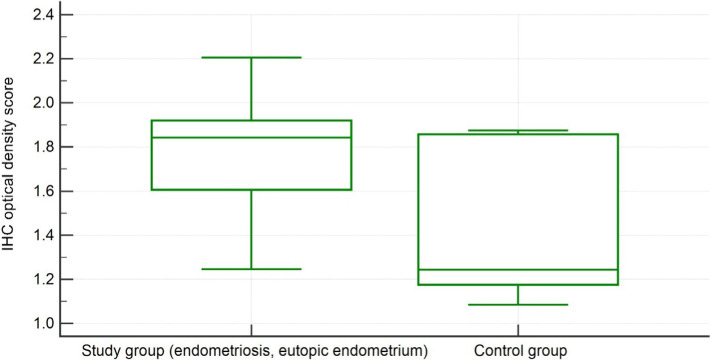
MMP-2 IHC optical density score comparison between group 1 (endometriosis, eutopic endometrium) and group 2 (the control group).

The measurement results of the IHC optical density score of immunohistochemical staining of MMP-2 in human eutopic endometrium obtained from patients with endometriosis and patients without endometriosis are represented in Figure 1. The IHC optical density score of MMP-2 in eutopic endometrium obtained from women with endometriosis was significantly higher (1.7783 ± 0.22 IHC optical density score) than in the eutopic endometrium obtained from women without endometriosis (1.41 ± 0.34, p = 0.0017).

As for the comparison of MMP-2 expression between ectopic endometrium in group 1 (with endometriosis) and eutopic endometrium in group 2 (control group), there was a significant difference between them (1.82 ± 0.27 IHC optical density score vs. 1.41 ± 0.34 in the control group, p = 0.001).

**Figure 2:**
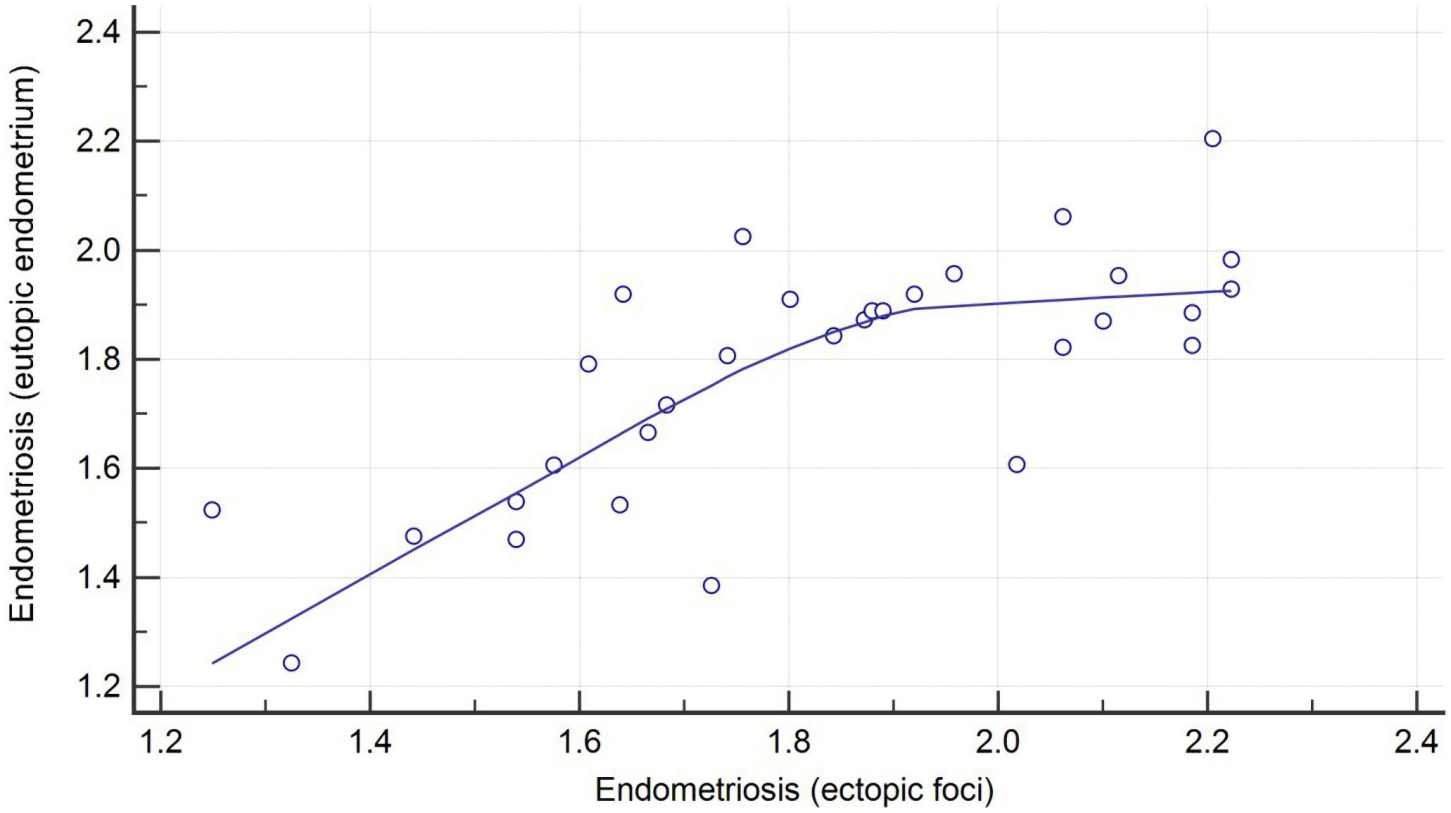
Correlation of MMP-2 IHC optical density between eutopic endometrium and ectopic endometriosis foci (correlation coefficient r = 0.74).

The correlation in the group 1 between ectopic and eutopic IHC optical density score was obtained. It showed a high score with r = 0.74, which indicates a strong correlation between the expression of MMP-2 in eutopic and ectopic endometrium (Figure 2).

**Figure 3:**
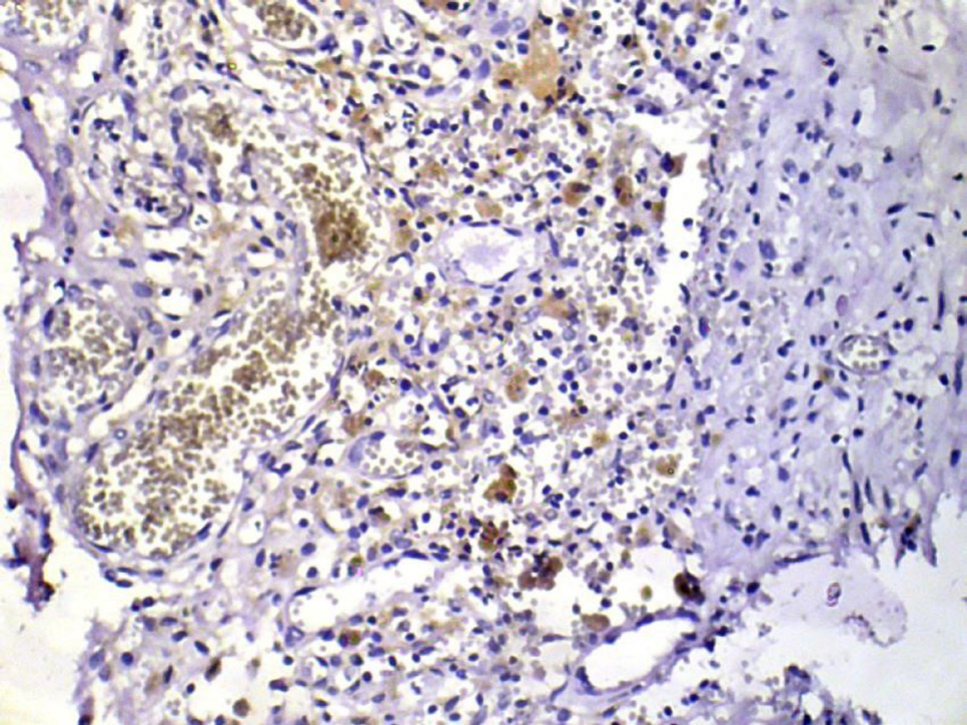
The visualization of immunohistochemical staining of MMP-2 in ectopic endometriosis foci: a light microscopic view. Stained areas are located mainly in the stroma and under the epithelial layer (x400).

**Figure 4:**
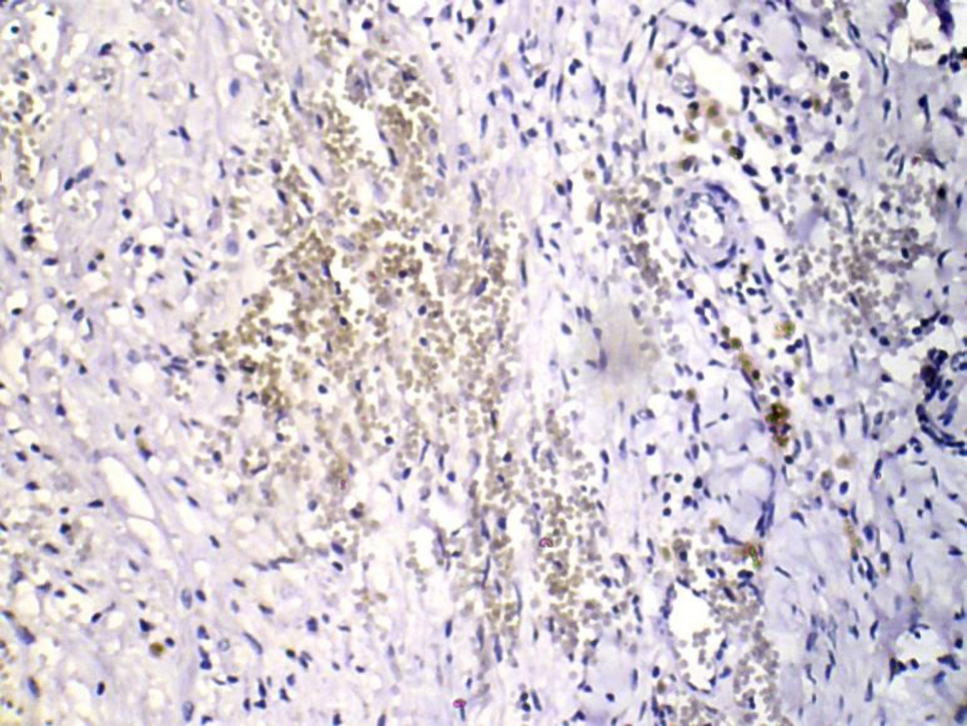
The visualization of immunohistochemical staining of MMP-2 in eutopic endometrium in endometriosis: a light microscopic view. Stained areas are located mainly in the stroma and around the vessels (x400).

On IHC images, MMP-2 stained areas were located mainly in the stroma and around the vessels (in eutopic endometrium) or directly under the epithelial layer (in ectopic endometrium) (Figure 3 and 4).

### MMP-9 expression

**Figure 5:**
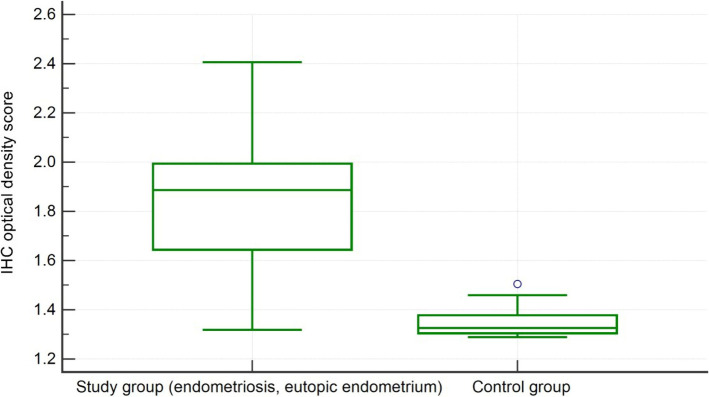
MMP-2 IHC optical density score comparison between group 1 (endometriosis, eutopic endometrium) and group 2 (the control group).

The measurement results of the IHC optical density score of immunohistochemical staining of MMP-9 in human eutopic endometrium obtained from patients with and without endometriosis are represented in Figure 5.

The IHC optical density score of MMP-9 in eutopic endometrium obtained from women with endometriosis was significantly higher (1.85 ± 0.26 IHC optical density score) than that in the eutopic endometrium obtained from women without endometriosis (1.3528 ± 0.067, p = 0.001). This indicates a significantly higher activity of MMP-9 in patients with endometriosis.

When comparing MMP-9 expression between ectopic endometrium in group 1 (with endometriosis) and eutopic endometrium in group 2 (control group), a significant difference between them was established (1.87 ± 0.30 IHC optical density score vs. 1.35 ± 0.06 in the control group, p < 0.001).

**Figure 6:**
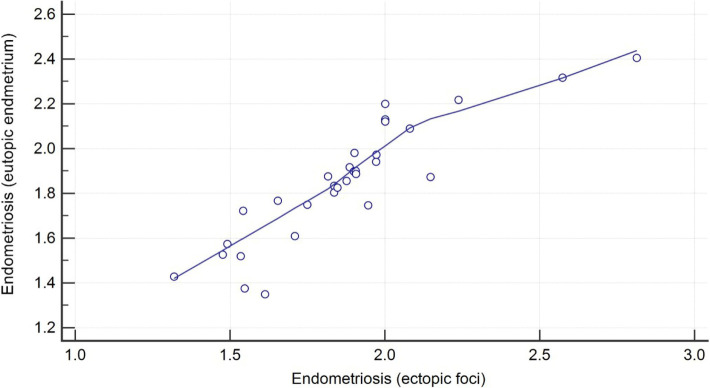
Correlation of MMP-9 IHC optical density between eutopic endometrium and ectopic endometriosis foci (correlation coefficient r = 0.88).

The correlation determined in group 1 between ectopic and eutopic IHC optical density score showed a high score with r = 0.88, which indicates a strong correlation between expression of MMP-9 in eutopic and ectopic endometrium as in case of MMP-2 (Figure 6).

**Figure 7:**
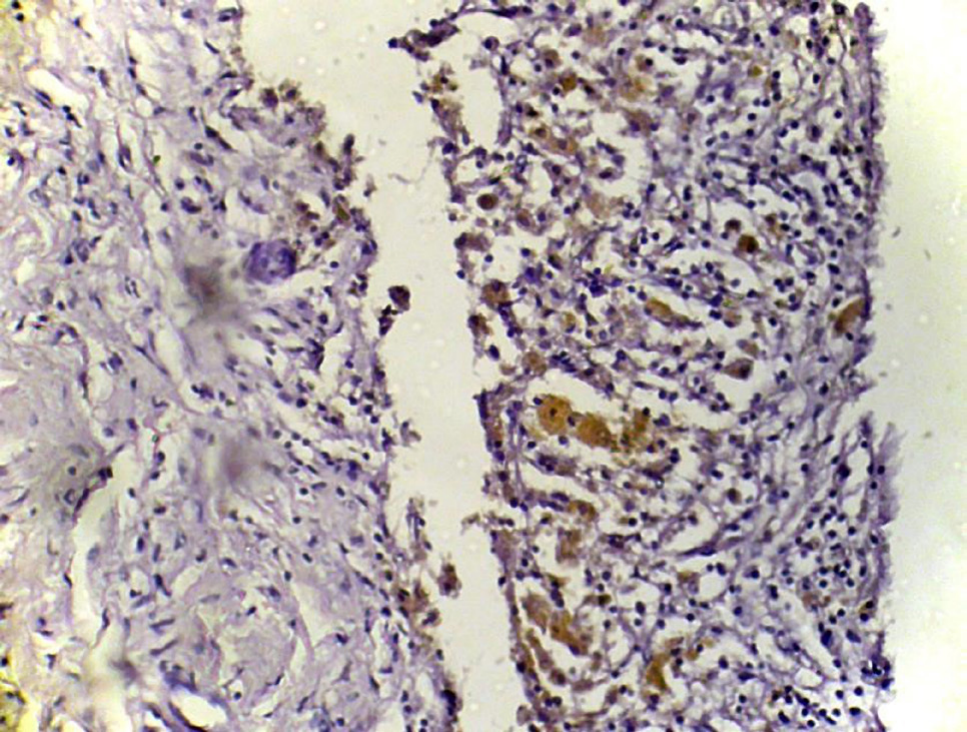
The visualization of immunohistochemical staining of MMP-9 in ectopic endometriosis foci: a light microscopic view. Stained areas are located in stroma, subepithelial and epithelial layer (x400).

**Figure 8:**
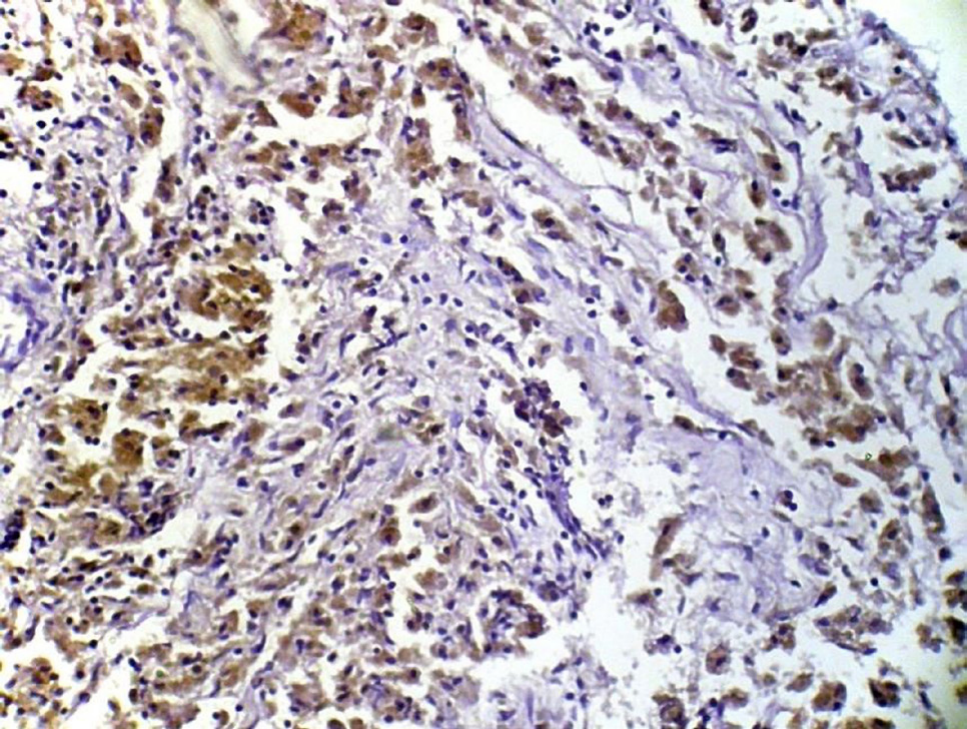
The visualization of immunohistochemical staining of MMP-9 in eutopic endometrium in endometriosis: a light microscopic view. Stained areas are located in the stroma and around the vessels (x400).

On IHC images, MMP-9 stained areas were located in the stroma and around the vessels (in eutopic endometrium) or in the sub-epithelial and epithelial layer (in ectopic endometrium) (Figure 7 and 8).

## Discussion

Matrix metalloproteinases (MMPs) are a family of endopeptidases playing a specific role in the degradation and turnover of the extracellular matrix (ECM). These zinc-dependent enzymes, including collagenases, gelatinases and stromelysins, are capable of degrading all the components of the ECM. Tissue inhibitors of metalloproteinases (TIMPs), which affect normal and pathologic matrix remodeling, regulate the activity of MMPs [29, 30]. MMP is supposed to enable the endometriotic tissue to be digested into the peritoneal ECM and underlying connective tissue. Endometrial remodeling and MMP expression are well known to occur during the proliferative and menstrual phases of the cycle, and that progesterone is a strong suppressor of MMPs [31]. The production of MMPs and their inhibitors takes place in the endometrial stroma and epithelium, as well as in polymorphic mononuclear leukocytes [32]. 

Taking into account the fact that the production of MMPs (as well as their inhibitors) takes place in the endometrial stroma, according to our findings, we suggest a hypothesis that MMPs activity in eutopic endometrium could correlate with the activity in ectopic foci of endometriosis. This peculiarity could improve the chances of non-invasive early diagnostics of endometriosis. This statement is also based on the opinion of Chung et al., who considers that increased proteolytic activity of the endometrial and endometriotic tissue may be one of the reasons for the invasive properties of the endometrium [33]. On this basis, we can assume that the endometrium could be a predominant source of MMP-9, as well as of MMP-2.

In the current study, we demonstrate that the expression of endometrium MMP-2 and MMP-9 are increased in patients with endometriosis. Similar conclusions were expressed by Meng-Hsing Wu et al. [34]. They found that MMP-9 plays a pivotal role in the peritoneal macrophage’s ability to degrade the basement membrane and, thus, its capability of phagocytosis. Liu, H et al. claim that plasma MMP-9 directly correlates with the severity of endometriosis and could be used as a biochemical marker [35]. However, we found only a few studies in which authors investigate MMP-2 expression in endometriosis [36].

Our study has particular strengths. First of all, we have established a correlation between ectopic and eutopic endometrium MMPs expression, which could help in the future in the early non-invasive diagnostic of endometriosis. Second, immunohistochemistry, a simple, reliable and informative method, has been used, which resulted in good visualization and the possibility of extensive use. Regarding the limitations of the studies conducted, it should be mentioned that we used a limited of research objects, which may slightly impair the accuracy of the results.

## Conclusions

Our study demonstrates a statistically higher expression of MMP-2 and MMP-9 both in ectopic and eutopic endometrium samples compared to samples taken from patients without endometriosis. A strong correlation between the expression of the above-mentioned MMPs in ectopic and eutopic endometrium might be of promising diagnostic value.

## Conflict of Interest

The authors declare that there is no conflict of interest.
